# Cognitive and clinical profiles in first-episode psychosis and their relationship with functional outcomes

**DOI:** 10.1192/bjp.2025.3

**Published:** 2026-03

**Authors:** Megan Cowman, Jo Hodgekins, Siân Lowri Griffiths, Emma Frawley, Karen O’Connor, David Fowler, Max Birchwood, Gary Donohoe

**Affiliations:** 1 Centre for Neuroimaging, Cognition & Genomics (NICOG), School of Psychology, University of Galway, Galway, Ireland; 2 Department of Clinical Psychology and Psychological Therapies, Norwich Medical School, University of East Anglia, Norwich, UK; 3 Institute for Mental Health, University of Birmingham, Birmingham, UK; 4 RISE Early Intervention in Psychosis Service, South Lee Mental Health Service, Cork, Ireland; 5 Psychology Department, University of Sussex, Brighton, UK; 6 Warwick Medical School, University of Warwick, Coventry, UK

**Keywords:** Psychosis, cognition, social cognition, cluster analysis, functional outcome

## Abstract

**Background:**

While cognitive impairment is a core feature of psychosis, significant heterogeneity in cognitive and clinical outcomes is observed.

**Aims:**

The aim of this study was to identify cognitive and clinical subgroups in first-episode psychosis (FEP) and determine if these profiles were linked to functional outcomes over time.

**Method:**

A total of 323 individuals with FEP were included. Two-step hierarchical and *k*-means cluster analyses were performed using baseline cognitive and clinical variables. General linear mixed models were used to investigate whether baseline cognitive and clinical clusters were associated with functioning at follow-up time points (6–9, 12 and 15 months).

**Results:**

Three distinct cognitive clusters were identified: a cognitively intact group (*N* = 59), a moderately impaired group (*N* = 77) and a more severely impaired group (*N* = 122). Three distinct clinical clusters were identified: a subgroup characterised by predominant mood symptoms (*N* = 76), a subgroup characterised by predominant negative symptoms (*N* = 19) and a subgroup characterised by overall mild symptom severity (*N* = 94). The subgroup with more severely impaired cognition also had more severe negative symptoms at baseline. Cognitive clusters were significantly associated with later social and occupational function, and associated with changes over time. Clinical clusters were associated with later social functioning but not occupational functioning, and were not associated with changes over time.

**Conclusions:**

Baseline cognitive impairments are predictive of both later social and occupational function and change over time. This suggests that cognitive profiles offer valuable information in terms of prognosis and treatment needs.

Cognitive impairment in psychotic disorders is prevalent, with approximately 80% of individuals exhibiting clinically significant impairment.^1^ Psychosis, from prodrome to chronic illness, is associated with cognitive impairment that is persistent, unresponsive to pharmacologic treatment and can hinder recovery at great financial, social and personal cost.^1–4^ For people living with psychosis, cognitive impairment can have significant impacts on functional recovery, engagement with treatment and quality of life more broadly.^5^ Importantly, both neurocognitive and social cognitive domains have been identified as significant longitudinal predictors of social and occupational function.^6^ As a result, cognition is becoming increasingly recognised as a critical treatment target, both in terms of the need for cognitive screening^7^ and cognitively focused interventions.^8^

## Cognitive and clinical profiles in psychosis

While some people with psychosis experience more pronounced cognitive impairment, others perform in line with the general population.^9–11^ Given this heterogeneity, researchers have attempted to define cognitive profiles in psychosis. Cognitive subgroups in both first-episode psychosis (FEP)^9–12^ and schizophrenia^13–17^ have been identified using cluster analysis, some based on degree of impairment and others based on selective impairments in specific domains, such as processing speed, attention, memory and executive function.^13,16,17^ Clusters were found to differ on socioeconomic status, premorbid IQ, negative symptom severity and functioning.^9–11^ Previous findings have also identified clinical subgroups in FEP.^18–21^ For example, Amoretti et al^18^ identified three distinct clinical clusters: groups characterised by overall mild symptoms, moderate negative symptoms and severe positive symptoms, respectively. Moreover, previous findings suggest that negative symptoms,^22^ positive symptoms^23^ and mood symptoms^24^ are predictive of functional outcomes. While previous findings suggest there are distinct cognitive subgroups in psychosis, results vary by number of clusters and nature of impairments. Social cognition has only been included in one study previously,^9^ despite its close relationship with neurocognition and functional outcomes.

## Aims

Given these findings, we aimed to investigate if participants could be grouped based on cognitive performance and clinical severity using cluster analyses, and if cluster membership at baseline was associated with functional outcomes at follow-up time points (6–9, 12 and 15 months). Identifying distinct cognitive and clinical subgroups within FEP can enhance our understanding of the illness to better inform prognosis and improve treatment outcomes through more personalised treatment. This may allow early intervention services (EISs) to identify people who are at increased risk for sustained functional disability or who are least likely to experience functional improvements.

## Method

### Participants

This study includes data from three different samples recruited from EIS sites in Ireland and the UK. Seventy-one participants were recruited as part of the PSYcHE study across two Irish EISs (Cork and Sligo). Ninety-eight participants were recruited from the EIS in Birmingham, UK. One hundred and fifty-four participants were recruited from four UK EISs as part of the National Institute for Health and Care Research (NIHR) SUPEREDEN trial. This subgroup of participants were those who had poor functioning after 12 months of EIS care (defined as engagement in less than 30 hours per week of structured activity on the Time Use Survey).^25^ Exclusion criteria for all samples included the following: (a) insufficient command of English language; (b) neurological disorder; (c) intellectual disability (also known as learning disability in UK health services); and (d) severe head injury. All participants had a confirmed diagnosis of FEP following a multidisciplinary team assessment according to *Diagnostic and Statistical Manual of Mental Disorders* 5th edition (DSM-V)^26^ or *International Statistical Classification of Diseases and Related Health Problems* 10th revision (ICD-10) criteria,^27^ were within 3 years of first contact with the EIS for a first episode of psychosis and were deemed clinically stable by the treating team. EISs provide a comprehensive range of interventions, including cognitive–behavioural therapy (CBT) for psychosis, family interventions, supported employment, assertive outreach case management and medical and psychopharmacological management. The authors assert that all procedures contributing to this work comply with the ethical standards of the relevant national and institutional committees on human experimentation and with the Helsinki Declaration of 1975, as revised in 2013. The EIS Birmingham and SUPEREDEN studies were approved by the National Research Ethics Service Committee in the Black Country, West Midlands (reference: 12/WM/0097). The PSYcHE study was approved by the Cork Teaching Hospitals Clinical Research Ethics Committee (reference: ECM 4 (I) 10/11/2020) and Sligo University Hospital Research Ethics Committee (reference: 845). As per ethical approval, parental consent was not required for participants aged 16–18. Young people over 16 are presumed to be capable of giving consent on their own behalf.^28^ All participants provided written informed consent.

### Measures

#### Social and occupational functioning

Social and occupational functioning was measured using two different measures. The PSYcHE sample had data for the Mental Illness Research, Education, and Clinical Center (MIRECC) version of the Global Assessment of Functioning (GAF) scale,^29^ including social and occupational subscales. The SUPEREDEN and EIS Birmingham samples had data for the Global Functioning: Social and Global Functioning: Role scales.^30^ Separate functioning scores for social and occupational functioning were converted to *z*-scores for each sample to standardise across measures. *Z*-score standardisation was used to harmonise scores across measures for comparability and did not include control comparisons. The scales are comparable in their measurement of social and occupational function and are described in further detail in Supplementary Section S1 available at https://doi.org/10.1192/bjp.2025.3. Functioning data were available at baseline, and at 6, 9, 12 and 15-month follow-up.

#### Clinical assessment

##### Duration of untreated psychosis (DUP)

Defined as the delay between onset of psychosis and initiation of treatment. DUP was measured in days and was clinician rated and/or gathered from information from clinical files for all samples.

##### Symptom severity

Two different measures of symptom severity were used. Two samples had data for the Positive and Negative Syndrome Scale (PANSS**)**.^31^ One sample had data for the Scale for the Assessment of Positive Symptoms (SAPS) and Scale for the Assessment of Negative Symptoms (SANS). Global total scores for the SAPS and SANS were converted to PANSS positive and negative symptom scores using an established method.^32^

##### Mood

Depression data were available for only two samples. One sample had data for the Beck Depression Inventory-II (BDI-II), a 21-item self-report measure.^33^ The other sample had data for the Patient Health Questionnaire (PHQ-9), a nine-item self-report measure of depression.^34^ PHQ-9 scores were converted to BDI-II scores using an established method.^35^

#### Cognitive assessment

##### Neurocognition

Verbal memory was assessed using the Logical Memory subtest (immediate) from the Wechsler Memory Scale Revised (WMS-R).^36^ Participants also completed subtests from the Wechsler Adult Intelligence Scale – Third Edition (WAIS-III):^37^ similarities and vocabulary (verbal comprehension) and matrix reasoning and block design (perceptual organisation). Raw scores on the subtests were converted to age standardised scores. The WAIS subtest scores were prorated (averaged and multiplied by number of subtests) to calculate an overall score as an indication of general cognitive function.

##### Social cognition

Two social cognition domains most commonly impaired in psychosis were included: the Theory of Mind (ToM) and emotion recognition. The ToM was assessed using the Picture Sequencing Task – false belief stories^38^ and Reading the Eyes in the Mind task.^39^ Both tasks are widely used in psychosis and demonstrate acceptable psychometric properties.^40,41^

Emotion recognition was assessed using the Mayer–Salovey–Caruso Emotional Intelligence Test – Perceiving Emotions (MSCEIT)^42^ and the Emotion Recognition Task (ERT) from the Cambridge Neuropsychological Test Automated Battery (CANTAB, 2019). Both tasks are widely used in psychosis and demonstrate acceptable psychometric properties.^43,44^ Scores for the ToM and ERT were converted to *z*-scores to standardise across measures.

### Statistical analyses

All data analyses were carried out with the Statistical Package for Social Sciences (SPSS version 27.0 for Windows; SPSS Inc., USA). Clinical and cognitive data were available for baseline only. Functioning data was available for baseline and 6–9, 12 and 15 months.

### Cluster analyses

For cluster analysis based on baseline cognitive performance; logical memory, WAIS subtests, emotion recognition *z*-scores and ToM *z*-scores were entered into the cluster analysis. For cluster analysis based on clinical symptoms, PANSS negative symptom score, PANSS positive symptom score and BDI-II mood score were entered into the cluster analysis. A two-phased clustering approach was used for each analysis. First, scores were entered into a hierarchical agglomerative cluster analysis with Ward’s linkage clustering and squared Euclidean distance. Ward’s method was chosen as it maximises the significance of difference between clusters.^45^ To determine the number of clusters to retain, the agglomeration coefficients and the dendrogram were examined. Next, a *k*-means cluster analysis was performed to confirm the hierarchical cluster solution. The stability of the cluster solution in response to variations in the data or clustering technique was tested by repeating the analysis over a range of scenarios. Acceptability was confirmed using discriminant function analysis to assess classification accuracy. A discriminant function plot was placed over the cluster solution to determine internal cluster quality.^46^ Cluster groups were compared on cognitive and clinical variables using analysis of variance (ANOVA) to confirm that clusters represented distinct subgroups. Clusters were also compared on demographic and functioning variables using ANOVA for continuous variables and Pearson’s *χ* ^2^ for categorical variables.

### General linear mixed model

To study longitudinal effects, general linear mixed effect modelling (GLMM) was performed to examine the relationship between cognitive and clinical clusters at baseline, and social and occupational functioning over time. For each outcome variable (social and occupational function) separate analyses were performed. Social and occupational functioning scores at four different time points (baseline and 6–9-, 12- and 15-month follow-up) were used as the dependent variables. Social functioning data were available for 307 participants at baseline, 165 at 6–9-month follow-up, 122 at 12-month follow-up and 111 at 15-month follow-up. Occupational functioning data were available for 290 participants at baseline, 136 at 6–9-month follow-up, 122 at 12-month follow-up and 86 at 15-month follow-up. Clusters (categorical variable) and time point (as a continuous variable) were entered as fixed effects and age and gender were entered as covariates. Interaction terms (time point by cognitive and clinical clusters) were added based on *a priori* hypotheses. This interaction term describes whether participants in different cluster groups change differently over time. Random effects included study sample and participant to control for variation between samples and individual participants, and the covariance between both. We explored different models with random effects and checked the Akaike information criterion (AIC) and Schwarz’s Bayesian information criterion (BIC) values and random variance accounted for.

## Results

### Description of sample

A total of 323 participants were included at baseline. Sociodemographic, clinical, functioning and cognitive characteristics are presented in Supplementary Table S1 (see Supplementary Table S2 for post hoc analyses). In terms of social and occupational function, the sample fell into the functionally impaired range for both social (mean 6.1, s.d. = 1.7) and occupational function (mean 4.4, s.d. = 2.7). Chi-squared tests revealed there were no significant associations between cognitive or clinical cluster membership and sample membership, or diagnostic category (see Supplementary Tables S3–6). Medication/drug use data was only available for the PSYcHE data-set (see Supplementary Section S2).

### Cluster solutions

Hierarchical clustering (Ward’s method) and *k*-means optimisation resulted in three distinct cognitive clusters based on 258 participants and three distinct clinical clusters based on 189 participants. Inspection of the dendrogram and agglomeration coefficient changes suggested a three-cluster solution was appropriate for both analyses and was found to be the most robust across a range of scenarios. The discriminant plots of the final solutions indicated relatively cohesive clusters with a concentration of cases around each centroid (see Supplementary Figs. S1 and S2). Linear discriminant function analysis showed significant differentiation and a high degree of classification accuracy in the three-cognitive-cluster model (92% cluster 1, 82% cluster 2, 90% cluster 3) and the three-clinical-cluster model (97% cluster 1, 95% cluster 2, 100% cluster 3).

Cognitive clusters were defined by degree of impairment, with a cognitively intact group (*N* = 59, approx. ½ s.d. above the general population mean), a moderately impaired group (*N* = 77, 1–2 s.d. below the general population mean) and a more severely impaired group (*N* = 122, >2 s.d. below the general population mean). Cognitive clusters were significantly different from one another on all cognitive, social and occupational functioning scores, and negative symptoms (see Table 1). See Supplementary Table S7 for post hoc analyses. The pattern of impairment in social cognition did not mirror that of neurocognitive impairment, whereby the moderately impaired group had better performance on both social cognitive measures than the intact group. However, this difference was not statistically significant. Both the intact and moderately impaired groups had significantly better performance on social cognitive measures than the severely impaired group. At final follow-up, the cognitively intact cluster had the highest percentage (63%) of participants who reached clinically significant functional recovery (i.e. above clinical cut-off score of 7) (see Table 2).

**Table 1:** Baseline demographic, clinical, functioning and cognitive characteristics for cognitive clusters

	Cluster 1 cognitively intact (*N* = 59)	Cluster 2 moderately impaired (*N* = 77)	Cluster 3 severely impaired (*N* = 122)	ANOVA
Demographic				
Male gender (*n*/%)	39 (66.1%)	51 (66.2%)	91 (74.6%)	*χ* ^2^ = 2.18, *p* = 0.34
Mean age (years; mean/s.d.)	24.5 (5.8)	25.2 (5.3)	26.5 (7.4)	*F* = 1.13, *p* = 0.32
Clinical				
DUP in days (median, range)	21 (2228)	41 (1168)	56 (5325)	*F* = 0.79, *p* = 0.46
Duration of illness (length of time in months from illness onset to baseline) (mean/s.d.)	19.2 (16.2)	19.8 (15.6)	17.7 (13.0)	*F* = 0.52, *p* = 0.59
Positive symptom severity (mean/s.d.)	12.1 (4.3)	12.9 (4.5)	12.8 (4.8)	*F* = 0.64, *p* = 0.53
Negative symptom severity (mean/s.d.)	12.2 (3.9)	13.8 (4.9)	16.4 (7.2)	*F* = 10.89, *p* < 0.001
BDI-II mood score (mean/s.d.)	21.3 (11.7)	19.5 (11.2)	16.5 (12.0)	*F* = 1.95, *p* = 0.15
Functioning (mean/s.d.)				
Social function	6.4 (1.4)	6.5 (1.8)	5.8 (1.7)	*F* = 4.99, *p* = 0.01
Occupational function	5.6 (2.6)	4.6 (2.9)	4.2 (2.6)	*F* = 4.84, *p* = 0.01
Cognition (mean/s.d.)				
Logical memory scaled score	10.8 (3.0)	7.0 (2.3)	5.3 (2.3)	*F* = 101.27, *p* < 0.001
WAIS perceptual reasoning subtest scaled score	12.0 (2.7)	8.8 (2.6)	7.2 (2.3)	*F* = 73.68, *p* < 0.001
WAIS verbal reasoning subtest scaled score	11.8 (2.7)	8.1 (2.5)	6.6 (2.2)	*F* = 96.36, *p* < 0.001
WAIS prorated sum of scaled scores	131.0 (23.1)	93.2 (23.1)	75.8 (20.4)	*F* = 126.78, *p* < 0.001
Theory of Mind *z*-score	0.4 (0.7)	0.7 (0.6)	−0.6 (0.9)	*F* = 78.66, *p* < 0.001
Emotion recognition *z*-score	0.6 (0.8)	0.7 (0.6)	−0.6 (0.9)	*F* = 80.31, *p* < 0.001

ANOVA, analysis of variance; DUP, duration of untreated psychosis; BDI-II, Beck Depression Inventory-II; WAIS, Wechsler Adult Intelligence Scale.

**Table 2:** Social and occupational functioning across time points for cognitive clusters

	Social function			Occupational function		
	% of cluster in fully functioning category at baseline	% of cluster in fully functioning category by 9 months	% of cluster in fully functioning category by final follow-up	% of cluster in fully functioning category at baseline	% of cluster in fully functioning category by 9 months	% of cluster in fully functioning category by final follow-up
**Cluster 1 intact**	48.2% (27/56)	62.1% (18/29)	63.4% (26/41)	36.4% (20/55)	44% (11/25)	62.9% (22/35)
Mean (s.d.)	6.0 (1.4)	6.7 (1.6)	6.6 (1.4)	4.2 (2.6)	6.4 (2.8)	6.6 (2.5)
**Cluster 2 moderately impaired**	54.7% (41/75)	43.9% (18/41)	50% (29/58)	33.3% (24/72)	43.8% (14/32)	50% (25/50)
Mean (s.d.)	6.3 (1.9)	6.4 (1.7)	6.0 (1.9)	4.2 (2.8)	5.9 (3.0)	5.8 (3.0)
**Cluster 3 severely impaired**	34.8% (40/115)	34.6% (18/52)	41.4% (41/99)	24.5% (27/110)	34.8% (16/46)	28.7% (27/94)
Mean (s.d.)	5.6 (1.7)	5.9 (1.7)	5.9 (1.6)	3.8 (2.3)	4.8 (2.4)	4.3 (2.3)

For clinical cluster analyses, the final cluster solution comprised three groups with distinct clinical profiles. Cluster 1 was characterised by predominant mood symptoms (*N* = 76), cluster 2 was characterised by predominant negative symptoms (*N* = 19) and cluster 3 was characterised by overall mild symptoms (*N* = 94). Clinical clusters were significantly different from one another on all clinical variables, social but not occupational functioning scores and all cognitive variables except ToM scores (see Table 3). See Supplementary Table S8 for post hoc analyses. At final follow-up, the predominant mood symptom cluster had the highest percentage (41%) of participants who reached clinically significant functional recovery (see Table 4).

**Table 3:** Baseline demographic, clinical, functioning and cognitive characteristics for clinical clusters

	Cluster 1 predominant mood symptoms (*N* = 76)	Cluster 2 predominant negative symptoms (*N* = 19)	Cluster 3 mild symptoms (*N* = 94)	ANOVA
Demographic				
Male gender (*n*/%)	47 (61.8%)	18 (94.7%)	66 (70.2%)	*χ* ^2^ = 7.8, *p* = 0.02
Mean age (years; mean/s.d.)	28.6 (7.0)	25.3 (4.0)	26.9 (7.8)	*F* = 0.39, *p* = 0.68
Clinical				
DUP in days (median, range)	46 (5325)	60 (1530)	61 (3561)	*F* = 1.44, *p* = 0.24
Duration of illness (length of time in months from illness onset to baseline) (mean/s.d.)	25.2 (17.3)	28.9 (13.7)	23.5 (12.6)	*F* = 1.09, *p* = 0.34
Positive symptom severity (mean/s.d.)	15.7 (5.1)	15.8 (5.5)	11.2 (3.7)	*F* = 23.71, *p* < 0.001
Negative symptom severity (mean/s.d.)	14.4 (3.7)	27.3 (5.1)	13.0 (4.2)	*F* = 95.64, *p* < 0.001
BDI-II mood score (mean/s.d.)	30.7 (6.4)	14.9 (8.8)	11.1 (6.3)	*F* = 186.65, *p* < 0.001
Functioning (mean/s.d.)				
Social function	5.4 (1.7)	4.9 (2.0)	6.3 (1.7)	*F* = 7.44, *p* < 0.001
Occupational function	3.7 (2.3)	2.9 (2.1)	4.1 (2.8)	*F* = 1.73, *p* = 0.18
Cognition (mean/s.d.)				
Logical memory scaled score	7.5 (3.7)	4.9 (2.7)	7.4 (3.2)	*F* = 4.71, *p* = 0.01
WAIS perceptual reasoning subtest scaled score	9.0 (3.1)	6.8 (2.1)	9.4 (3.3)	*F* = 4.88, *p* = 0.01
WAIS verbal reasoning subtest scaled score	8.6 (3.4)	5.9 (2.3)	8.3 (3.0)	*F* = 5.60, *p* = 0.01
WAIS prorated sum of scaled scores	96.8 (32.2)	70.2 (18.2)	98.0 (31.0)	*F* = 6.12, *p* = 0.00
Theory of mind *z*-score	0.1 (0.9)	−0.1 (0.7)	0.2 (0.9)	*F* = 0.78, *p* = 0.46
Emotion recognition *z*-score	0.4 (1.0)	−0.5 (0.8)	0.1 (1.1)	*F* = 6.76, *p* = 0.00

ANOVA, analysis of variance; DUP, duration of untreated psychosis; BDI-II, Beck Depression Inventory-II; WAIS, Wechsler Adult Intelligence Scale.

**Table 4:** Social and occupational functioning across time points for clinical clusters

	Social function			Occupational function		
	% of cluster in fully functioning category at baseline	% of cluster in fully functioning category by 9 months	% of cluster in fully functioning category by final follow-up	% of cluster in fully functioning category at baseline	% of cluster in fully functioning category by 9 months	% of cluster in fully functioning category by final follow-up
**Cluster 1 predominant mood symptoms**	28.4% (21/74)	37.5% (24/64)	38% (19/50)	12.3% (8/65)	25.5% (12/47)	40.5% (15/37)
Mean (s.d.)	5.4 (1.7)	5.6 (1.9)	5.7 (1.6)	3.4 (2.1)	5.0 (2.6)	5.3 (2.7)
**Cluster 2 predominant negative symptoms**	21.1% (4/19)	7.1% (1/14)	0% (0/12)	5.9% (1/17)	22.2% (2/9)	33.3% (2/6)
Mean (s.d.)	4.7 (1.8)	4.7 (1.4)	4.8 (1.3)	2.8 (1.8)	3.4 (2.6)	3.6 (2.4)
**Cluster 3 mild symptoms**	47.9% (45/94)	55.6% (40/72)	47.7% (31/65)	20.5% (18/88)	40.0% (26/65)	27.1% (16/59)
Mean (s.d.)	6.2 (1.7)	6.7 (1.3)	6.4 (1.6)	3.5 (2.2)	5.1 (2.7)	4.7 (2.6)

An additional exploratory cluster analysis was carried out to investigate if distinct clusters could be identified based on both cognitive and clinical variables combined based on a reduced sample size of 136 participants for those who had relevant data (see Supplementary Section S3 and Tables S9 and S10 for results).

### GLMM

GLMM analysis showed significant differences in cognitive clusters for social (*F* = 8.4, *p* < 0.001) and occupational (*F* = 3.9, *p* = 0.02) functioning over time. For social functioning, the slope of the cognitively intact cluster (*β* = 0.021) was significantly different from the slope of the moderately impaired cluster (*β* = −0.010) (*t* = 2.462, 95% CI [0.007–0.058], *p* = 0.014) but not the severely impaired cluster. The slope of the severely impaired cluster (*β* = 0.018) and the moderately impaired cluster were also significantly different (*t* = 2.749, 95% CI [0.008–0.051], *p* = 0.006). The intact cluster showed the largest improvement in social functioning scores of the three clusters, albeit with a relatively small increase of 0.6, the impaired cluster showed a small increase of 0.3 and the moderately impaired cluster showed a small decrease in function (−0.3). Social functioning for the severely and moderately impaired clusters converged over time, and were comparable at final follow-up.

For occupational function, the slope of the intact cluster (*β* = 0.050) was significantly different from the severely impaired cluster (*β* = 0.016) (*t* = −2.014, 95% CI [−0.0064 to 0.001, *p* = 0.04), but not the moderately impaired cluster (*β* = 0.042). The intact cluster showed the largest improvement in occupational functioning score with an increase of 2.4, the moderate cluster showed a smaller increase of 1.6 and the impaired cluster showed the smallest increase of 0.5.

A significant difference in clinical clusters was found for social (*F* = 6.48, *p* = 0.002) but not occupational function. The slope for changes in social functioning did not differ significantly across clinical clusters.

### Post hoc exploratory analyses

Two samples (PSYcHE and SUPEREDEN) included different assessments of premorbid function. Although we were unable to combine these scores to include in our main analyses, we conducted correlation analyses within each individual sample to explore the associations between premorbid function and social and occupational function, clinical variables and cognitive performance. The results of this are reported in Supplementary Tables S11 and S12.

## Discussion

### Summary of findings

To the authors’ knowledge, this is the largest study to use cluster analysis to identify cognitive and clinical profiles within FEP samples, with the inclusion of measures of social cognition. In addition, this is the first study to evaluate the predictive value of cognitive and clinical clusters at baseline for follow-up functioning, with multiple follow-up time points. Our analyses identified three distinct cognitive subgroups: one with intact cognitive function, one with moderate cognitive impairments and one with more significant cognitive impairments. Our results are consistent with previous findings suggesting that subgroup classification is based on degree of impairment.^9^ Some 23% of the sample fell within the cognitively intact subgroup, and almost half in the more severely impaired subgroup. This is in line with previous studies that report approximately 19–28% of individuals with psychosis have intact cognitive function,^12,47^ but lower than some previous studies.^9,11,17^ However, two of these studies included healthy controls in their cluster analyses.

Importantly, cognitive subgroup membership was significantly associated with follow-up social and occupational functioning, and with differences in changes in functioning over time. While there was an overall increase for the total sample in occupational and social function, the two lower performing subgroups were less likely to achieve functional recovery at follow-up compared with the subgroup with intact cognition. The more severely impaired cognitive subgroup also had the highest level of negative symptom severity. The clinical cluster analysis results mirrored this finding, whereby the predominant negative symptoms cluster had the lowest level of functioning at baseline and follow-up, although this group consisted of only 19 participants. A previous review of symptomatology and cognitive impairment reported that cognitive deficits are associated with negative and disorganised dimensions rather than positive and depressive dimensions.^48^ In line with previous findings,^49,50^ the current findings suggest people who experience both cognitive impairment and more severe negative symptoms are at increased risk for prolonged functional impairment.

### Limitations

Some limitations of the current study are noted. For cognitive variables, only measures of general cognitive function, memory, ToM and emotion recognition were available for all data-sets. Previous studies typically used a larger neuropsychological battery, and could be able to better detect more subtle differences across specific domains. The absence of a measure of processing speed in particular, which some authors have argued is more sensitive to cognitive difficulties in psychosis than other cognitive measures,^51^ may have affected the nature of reported groupings. In addition, while widely used in psychosis research, the social cognition measures included are not as well evaluated in terms of psychometric properties as other measures.^52^ Similarly, previous studies investigating clinical subtypes included a wider variety of symptom dimensions. The negative symptom measures included are limited in that they may not fully capture more nuanced aspects, and are unable to distinguish specific subdomains. More detailed negative symptom assessment could allow for a better understanding of distinct clinical profiles in FEP. In addition, the sample size for the clinical cluster analysis was more limited, and thus these findings should be interpreted with caution. However, our findings suggest it is possible to identify clinically useful subgroups based on a smaller assessment battery. The small number of participants included with affective psychosis meant we were unable to properly evaluate differences based on diagnosis. However, previous studies using cluster analysis have shown that there is no significant association between cluster membership and diagnosis, and different diagnoses are represented across cognitive clusters.^9,17^ Another limitation is the combination of samples from different studies, at varying stages of engagement with the EIS. Data in the current study were gathered from three separate but related studies, with slightly different study criteria. While all researchers and clinicians were experienced and received standardised training in assessment, the multi-site nature of the studies included introduces variability. In addition, raters at follow-up were not blind to the clinical and cognitive ratings at baseline. Notwithstanding these limitations, the combination of samples may contribute to the results being more generalisable across the broad spectrum of cognitive function, clinical severity and social and occupational function in psychosis. However, our sample was limited in terms of diversity, which limits the generalisability of the findings cross-culturally. Finally, we were unable to investigate the effects of potential confounding variables such as antipsychotic medication use, cannabis use and premorbid functioning.

### Future directions

The current findings highlight important questions for future research. Given the evident heterogeneity in cognitive function in FEP, it would be useful to investigate cognitive clusters in clinical high-risk (CHR) individuals to determine if cognitive subgroups could be useful in determining prognosis. The stability of cognitive clusters across the course of illness and the association with functioning could be explored to understand how this might change depending on the stage of illness. In addition, the predictive value of cognitive profiles for long-term functional outcomes could be evaluated at service entry. It would also be useful to determine if baseline cognitive clusters were associated with follow-up negative symptoms, as previous studies have shown that individuals who experience higher cognitive performance also have a greater reduction in the severity of negative symptoms.^53^ In addition, newer tools, such as the Brief Negative Symptom Scale (BNSS), which offer better understanding of specific subdomains, may lead to better understanding of the complex relationship among cognitive impairment, negative symptoms and function. Given the demand cognitive assessment places on both staff of EISs and patients, it would be interesting to evaluate the utility of a short assessment battery compared to a wider battery including multiple domains for identifying cognitive profiles. Our previous findings suggest a brief cognitive assessment could be used to predict broader neurocognitive performance in a manner practical for screening use.^54^ From an EIS perspective, it would be useful to evaluate if cognitive profiles could be identified based on a shorter assessment battery, if these profiles reliably represented overall cognitive function and if they reliably predict functional outcomes over time.

These findings suggest that distinct cognitive profiles are evident in FEP, and predict follow-up functioning. The evidence of variable functional trajectories suggests a need for more tailored interventions to optimise longer-term outcomes. Functional recovery, even in those with moderate symptoms and intact cognition, was not particularly high across the sample as a whole. These findings highlight the importance of focusing on functioning, and interventions to support this. The subgroup who are most severely impaired might benefit from more cognitively focused interventions such as cognitive remediation therapy (CRT).^8^ The group with higher levels of negative symptoms might benefit from an intervention with a focus on assertive outreach, such as social recovery therapy.^55^ People who experience cognitive impairment and high negative symptom severity might benefit from an intervention that combines both CRT and social recovery therapy.^56^ These findings also highlight the usefulness of cognitive screening in EISs. While assessment of clinical symptoms forms part of routine assessment, cognitive assessment does not. Our findings suggest it is possible to profile people based on a short cognitive assessment battery without the need for an extended battery across multiple cognitive domains. Identification of cognitive impairments early in the course of illness may be useful for guiding treatment options.

## Supporting information

Cowman et al. supplementary material Cowman et al. supplementary material

## Data Availability

The data that support the findings of this study are not publicly available owing to ethical restrictions and regulation of the privacy of participants’ data, but are available from the corresponding author on reasonable request (G.D.).

## Supplementary material

The supplementary material for this article can be found at https://doi.org/10.1192/bjp.2025.3
