# Supplementary material for: Cognitive and clinical profiles in first-episode psychosis and their relationship with functional outcomes
Source: Br J Psychiatry. 2025 Mar 26;228(3):236–43. doi: 10.1192/bjp.2025.3 (PMC12912872; doi:10.1192/bjp.2025.3)
Supplement: Cowman et al. supplementary material [file S0007125025000030sup001.docx]

**Supplementary Information**

**S1. Detailed description of functioning measures.**

Two functioning measures were used across the samples included in the current study; the Mental Illness Research, Education, and Clinical Center (MIRECC) version of the Global Assessment of Functioning (GAF) scale^1^, and the Global Functioning Scale (GFS): Social and Global Functioning Scale: Role.^2^ Both scales have an occupational function and social function subscale scores. For both scales, lower scores indicate more impairment in that domain, and higher scores indicate better occupational and social functioning. The MIRECC GAF scores range from 0 to 100, and the GFS scores range from 0 to 10. For the MIRECC GAF, a score of less than 70 suggests impaired functioning. For the GFS, a score of less than 7 suggests impaired functioning. Both scales are interviewer-rated and ratings are based on a thorough assessment of the participant’s functional status. Both scales are rated based on current level of functioning. Both scales include anchor points associated with qualitative descriptions of functioning related to frequency and quality of work, school and caregiving for occupational function, and interpersonal conflict, relationships and ability to interact socially for social functioning. For social functioning subscales on both measures, the emphasis is on social contact/interactions with people other than family members and or/ professionals, unless these are the only interpersonal contacts a person has. The MIRECC GAF and GFS subscales are divided into ten equal intervals and include criteria for scoring within each interval. Both scales have a cut off score of 7 or 70 to indicate a good level of functioning.

The main difference between the scales is that the GFS includes a greater level of detail between the scoring intervals, whereas the MIRECC GAF groups the intervals into broader categories so is slightly more ambiguous. For example, the GFS includes the following ratings: extreme role dysfunction, inability to function, marginal ability to function, major impairment, serious impairment, moderate, mild impairment, good functioning, above average functioning and superior functioning. The MIRECC GAF includes the following ratings: fully functional (70 and above), borderline (50-70), dysfunctional (50 and below). This could lead to different scoring trends between measures which is a limitation of combining scores. However, in almost all types of functioning measures commonly used in research and practice, there is inherent subjectivity and bias. While raters are trained to score in an objective and consistent manner, this subjectivity and bias is somewhat unavoidable. Both scales have been validated for use in research with participants with psychosis, with established reliability and validity for assessing social and occupational function.^1,2^ All researchers and clinicians in the current study were trained in the administration and scoring of functioning assessments.

**References**

1 Niv N, Cohen AN, Sullivan G, Young AS. The MIRECC Version of the Global Assessment of Functioning Scale: Reliability and Validity. *PS* 2007; **58**: 529–535.

2 Cornblatt BA, Auther AM, Niendam T, Smith CW, Zinberg J, Bearden CE *et al.* Preliminary Findings for Two New Measures of Social and Role Functioning in the Prodromal Phase of Schizophrenia. *Schizophrenia Bulletin* 2007; **33**: 688–702.

Table 1. Baseline demographic, clinical, functioning and cognitive characteristics for total and individual samples.

|  | Total sample | PSYcHE sample  (N=71) | Birmingham sample (N=98) | SUPEREDEN sample (N=154) | ANOVA/ Chi-  Squared |
| --- | --- | --- | --- | --- | --- |
| Demographic |  |  |  |  |  |
| Male sex (n/ %) | 229 (70.9%) | 43 (60.6%) | 70 (71.4%) | 116 (75.3%) | χ^2^=5.15, *p*=0.076 |
| Mean age (mean/SD/  range) | 26.0 (6.6)  (16-56) | 30.4 (9.1)  (18-56) | 23.6 (4.7)  (16-35) | 25.3 (4.9)  (16-35) | *F*=25.79, *p*<0.001 |
| Ethnicity (n/%) |  |  |  |  | χ^2^=94.31, *p<*0.001 |
| *Caucasian* | 198 (61.3%) | 64 (90.1%) | 42 (42.9%) | 92 (59.7%) |  |
| *Asian* | 51 (15.8%) | 0 (0%) | 34 (34.7%) | 17 (11%) |  |
| *Black* | 25 (7.7%) | 4 (5.6%) | 14 (14.3%) | 7 (4.5%) |  |
| *Other* | 18 (5.6%) | 3 (4.2%) | 8 (8.2%) | 7 (4.5%) |  |
| Education level (n/%) |  |  |  |  | χ^2^=89.80, *p*<0.001 |
| *Primary level or less* | 28 (8.7%) | 5 (7%) | 9 (9.2%) | 14 (9.3%) |  |
| *Secondary level* | 154 (41.5%) | 44 (62%) | 36 (36.7%) | 54 (35.8%) |  |
| *Post-secondary further education* | 43 (13.3%) | 0 (0%) | 36 (36.7%) | 7 (4.6%) |  |
| *Third level* | 114 (35.3%) | 22 (31%) | 17 (17.3%) | 75 (49.7%) |  |
| Clinical |  |  |  |  |  |
| Diagnosis (n/%) |  |  |  |  | χ^2^=18.85, *p*<0.001 |
| *Non-affective psychosis* | 304 (94.1%) | 62 (87.3%) | 88 (89.8%) | 154 (100%) |  |
| *Affective psychosis* | 19 (5.9%) | 9 (12.7%) | 10 (10.2%) | 0 (0%) |  |
| DUP in days (median, range) | 55 (5325) | 21 (2920) | 54 (1738) | 72 (5325) | *F*=2.91, *p=*0.056 |
| Duration of illness (length of time in months from illness onset to baseline)  (mean/SD) | 19.48 (14.35) | 12.76 (16.18) | 13.23 (10.05) | 26.82 (12.29) | *F*=45.97, *p*<0.001 |
| Positive symptom severity (mean/SD) | 13.0 (4.9) | 11.6 (2.7) | 12.3 (4.9) | 14.0 (5.3) | *F*=7.29, *p*<0.001 |
| Negative symptom severity (mean/SD) | 14.7 (6.0) | 12.0 (3.7) | 14.4 (6.5) | 16.1 (6.1) | *F*=11.12,  *p*<0.001 |
| BDI-II mood score (mean/SD) | 19.3 (11.5) | 21.2 (9.9) | unavailable | 18.8 (11.9) | *F*=1.41, *p=*0.237 |
| Functioning (mean/SD) |  |  |  |  |  |
| Social function | 6.1 (1.7) | 6.9 (1.6) | 6.4 (1.5) | 5.5 (1.8) | *F*=19.46, *p*<0.001 |
| Occupational function | 4.4 (2.7) | 5.9 (2.9) | 5.0 (2.8) | 3.3 (2.1) | *F*=25.78, *p*<0.001 |
| Cognition (mean/SD) |  |  |  |  |  |
| Logical memory scaled score | 7.1 (3.2) | 6.8 (2.8) | 6.9 (3.1) | 7.3 (3.5) | *F*=0.69, *p=*0.502 |
| WAIS perceptual reasoning subtest scaled score | 8.8 (3.1) | 10.9 (3.0) | 8.2 (2.8) | 8.1 (3.0) | *F*=24.84, *p*<0.001 |
| WAIS verbal reasoning subtest scaled score | 8.1 (3.1) | 9.4 (2.9) | 7.8 (3.0) | 7.7 (3.2) | *F*=7.40, *p*<0.001 |
| WAIS prorated sum of scaled scores | 93.3 (30.8) | 112.1 (28.8) | 87.6 (30.0) | 87.0 (29.9) | *F*=19.10, *p*<0.001 |
| Theory of Mind z score | -0.01 (1.00) | -0.03(1.00) | -0.22 (1.16) | 0.18 (0.80) | *F*=4.43  *p=*0.013 |
| Emotion recognition z score | 0.00 (1.00) | 0.00 (1.01) | -0.14 (0.92) | 0.12 (1.06) | *F*=1.84, *p=*0.161 |

Table 2. Post-hoc analyses for significant differences between samples.

| **Variable** | **Comparison*** | **Mean Difference** | **Standard error** | **p-value** | **95% CI** |
| --- | --- | --- | --- | --- | --- |
| **Age** | Sample A vs Sample B | 6.72 | 0.96 | <0.001 | 4.46 – 8.97 |
|  | Sample A vs Sample C | 5.01 | 0.92 | <0.001 | 2.85 – 7.16 |
|  | Sample B vs Sample C | -1.71 | 0.83 | 0.100 | -3.67 – 0.25 |
| **Duration of illness** | Sample A vs Sample B | -0.46 | 2.00 | 0.971 | -5.17 – 4.24 |
|  | Sample A vs Sample C | -14.06 | 1.86 | <0.001 | -18.43 - -9.68 |
|  | Sample B vs Sample C | -13.59 | 1.66 | <0.001 | -17.50 - -9.69 |
| **Positive symptoms** | Sample A vs Sample B | -0.61 | 0.77 | 0.704 | -2.43 – 1.20 |
|  | Sample A vs Sample C | -1.77 | 0.62 | 0.013 | -3.23 – 0.30 |
|  | Sample B vs Sample C | -1.71 | 0.83 | 0.100 | -3.67 – 0.25 |
| **Negative symptoms** | Sample A vs Sample B | -2.37 | 0.94 | 0.033 | -4.58 – -0.15 |
|  | Sample A vs Sample C | -4.04 | 0.87 | <0.001 | -6.09 – -2.00 |
|  | Sample B vs Sample C | -1.68 | 0.76 | 0.071 | -3.46 – 0.11 |
| **Social function** | Sample A vs Sample B | 0.48 | 0.28 | 0.193 | -0.17 – 1.13 |
|  | Sample A vs Sample C | 1.44 | 0.26 | <0.001 | 0.83 – 2.04 |
|  | Sample B vs Sample C | 0.96 | 0.21 | <0.001 | 0.45 – 1.46 |
| **Occupational function** | Sample A vs Sample B | 0.84 | 0.42 | 0.115 | -0.15 – 1.82 |
|  | Sample A vs Sample C | 2.56 | 0.40 | <0.001 | 1.62 – 3.49 |
|  | Sample B vs Sample C | 1.72 | 0.33 | <0.001 | 0.94 – 2.50 |
| **WAIS perceptual reasoning subtest scaled score** | Sample A vs Sample B | 2.75 | 0.46 | <0.001 | 1.67 – 3.82 |
|  | Sample A vs Sample C | 2.88 | 0.44 | <0.001 | 1.85 – 3.92 |
|  | Sample B vs Sample C | 0.14 | 0.40 | 0.935 | -0.79 – 1.07 |
| **WAIS verbal reasoning subtest scaled score** | Sample A vs Sample B | 1.55 | 0.48 | 0.004 | 0.41 – 2.69 |
|  | Sample A vs Sample C | 1.68 | 0.46 | <0.001 | 0.59 – 2.76 |
|  | Sample B vs Sample C | 0.13 | 0.42 | 0.949 | -0.86 – 1.12 |
| **WAIS prorated sum of scaled scores** | Sample A vs Sample B | 24.42 | 4.60 | <0.001 | 13.60 – 35.25 |
|  | Sample A vs Sample C | 25.08 | 4.38 | <0.001 | 14.76 – 35.40 |
|  | Sample B vs Sample C | 0.66 | 3.99 | 0.985 | -8.75 – 10.07 |
| **Theory of Mind z score** | Sample A vs Sample B | 0.19 | 0.15 | 0.432 | -0.17 – 0.56 |
|  | Sample A vs Sample C | -0.21 | 0.15 | 0.334 | -0.56 – 0.14 |
|  | Sample B vs Sample C | -0.40 | 0.14 | 0.009 | -0.72 – 0.08 |

*Sample A: PSYcHE, Sample B: Birmingham, Sample C: SUPEREDEN.

Table 3. Chi square analysis comparing cognitive clusters and samples.

|  | **PSYcHE sample**  **(N=60)** | **Birmingham sample (N=93)** | **SUPEREDEN sample (N=105)** |
| --- | --- | --- | --- |
|  | N (%) | N (%) | N (%) |
| **Cluster 1 intact (N=59)** | 18 (30.5%) | 16 (27.1%) | 25 (42.4%) |
| **Cluster 2 moderately impaired**  **(N=77)** | 16 (20.8%) | 23 (29.9%) | 38 (49.4%) |
| **Cluster 3 severely impaired**  **(N=122)** | 26 (21.3%) | 54 (44.3%) | 42 (34.4%) |

χ^2^ = 8.78, *p*=0.07

Table 4. Chi square analysis comparing clinical clusters and samples.

|  | **PSYcHE sample**  **(N=41)** | **SUPEREDEN sample (N=148)** |
| --- | --- | --- |
|  | N (%) | N (%) |
| **Cluster 1 predominant mood symptoms (N=76)** | 19 (25.0%) | 57 (75%) |
| **Cluster 2 predominant negative symptoms (N=19)** | 0 (0%) | 19 (100%) |
| **Cluster 3 mild symptoms**  **(N=94)** | 22 (23.4%) | 72 (76.6%) |

χ^2^ = 5.92, *p*=0.06

Table 5. Chi square analysis comparing cognitive clusters and diagnosis.

|  | **Non-affective psychosis**  **(N=60)** | **Affective psychosis (N=93)** |
| --- | --- | --- |
|  | N (%) | N (%) |
| **Cluster 1 intact (N=59)** | 55 (93.2%) | 4 (6.8%) |
| **Cluster 2 moderately impaired**  **(N=77)** | 73 (94.8%) | 4 (5.2%) |
| **Cluster 3 severely impaired**  **(N=122)** | 112 (91.8%) | 10 (8.2%) |

χ^2^=0.66, *p*=0.72

Table 6. Chi square analysis comparing clinical clusters and diagnosis.

|  | **Non-affective psychosis**  **(N=60)** | **Affective psychosis (N=93)** |
| --- | --- | --- |
|  | N (%) | N (%) |
| **Cluster 1 predominant mood symptoms (N=76)** | 74 (97.4%) | 2 (2.6%) |
| **Cluster 2 predominant negative symptoms (N=19)** | 19 (100%) | 0 (0%) |
| **Cluster 3 mild symptoms**  **(N=94)** | 91 (96.8%) | 3 (3.2%) |

χ^2^=0.63, *p*=0.73

**S2. Medication and drug use data for PSYcHE sample.**

The most frequently prescribed anti-psychotic medication were Olanzapine (N=20) and Aripiprazole (20), followed by Quetiapine (N=5), Risperidone (N=5), Clozapine (N=4), Amisulpride (N=3), Paliperidone (N=2) and Cariprazine (N=1). Four participants were prescribed two antipsychotics, and eleven participants were not taking antipsychotic medication. Defined daily dose (DDD) equivalent values for Olanzapine were calculated (M=10.06, SD=18.12). Self-reported drug use in the month prior to assessment was recorded. 12% (N=8/66) reported using cannabis in the month prior. Mean grams of cannabis consumed was 4.1 (SD=1.7). 4% (N=3/68) reported using other drugs in the month prior.


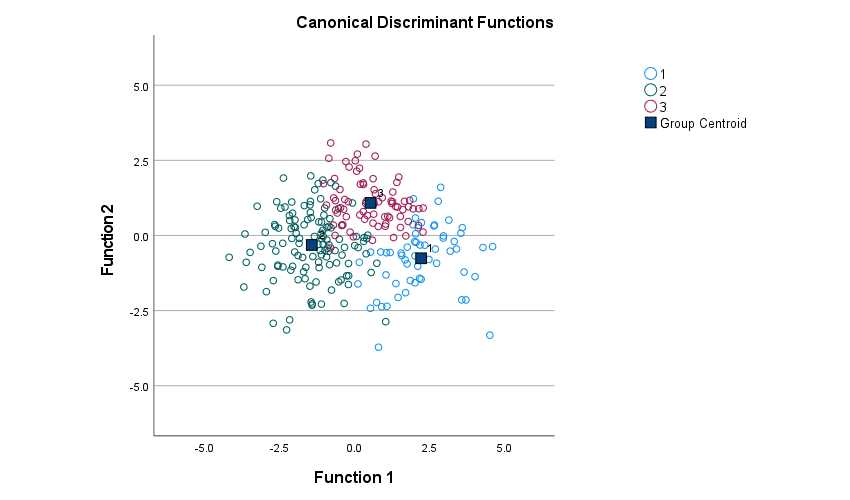


Figure 1. Discriminant plot of cognitive cluster solution.


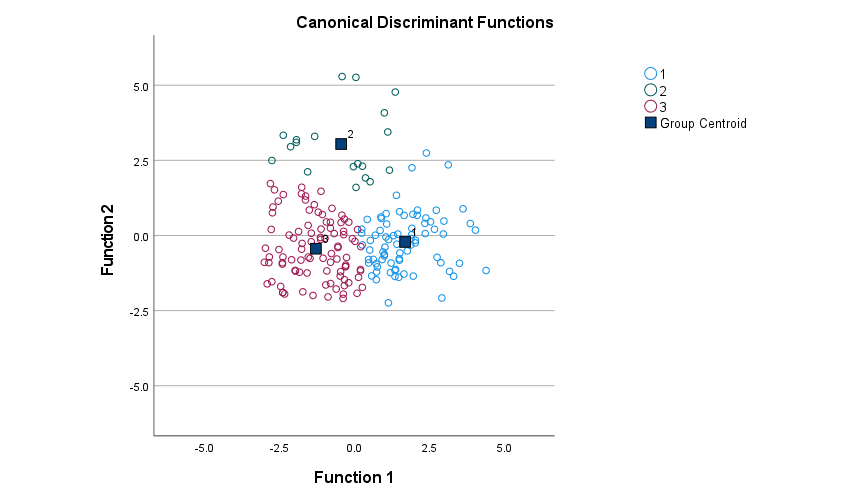


Figure 2. Discriminant plot of clinical cluster solution.

Table 7. Post-hoc analyses for significant differences between cognitive clusters.

| **Variable** | **Comparison*** | **Mean Difference** | **Standard error** | **p-value** | **95% CI** |
| --- | --- | --- | --- | --- | --- |
| **Negative symptoms** | Cluster 1 vs Cluster 2 | -1.60 | 1.05 | 0.281 | -4.06 – 0.87 |
|  | Cluster 1 vs Cluster 3 | -4.21 | 0.95 | <0.001 | -6.46 - -1.96 |
|  | Cluster 2 vs Cluster 3 | 2.61 | 0.88 | 0.009 | -4.69 - -0.54 |
| **Social function** | Cluster 1 vs Cluster 2 | -0.05 | 0.30 | 0.982 | -0.75 – 0.65 |
|  | Cluster 1 vs Cluster 3 | 0.65 | 0.27 | 0.05 | 0.00 – 1.29 |
|  | Cluster 2 vs Cluster 3 | 0.70 | 0.25 | 0.015 | 0.11 – 1.29 |
| **Occupational function** | Cluster 1 vs Cluster 2 | 0.97 | 0.48 | 0.107 | -0.16 – 2.10 |
|  | Cluster 1 vs Cluster 3 | 1.37 | 0.44 | 0.006 | 0.33 – 2.41 |
|  | Cluster 2 vs Cluster 3 | 0.40 | 0.41 | 0.587 | -0.56 – 1.36 |
| **Logical memory scaled score** | Cluster 1 vs Cluster 2 | 3.84 | 0.43 | <0.001 | 2.84 – 4.85 |
|  | Cluster 1 vs Cluster 3 | 5.57 | 0.40 | <0.001 | 4.65 – 6.49 |
|  | Cluster 2 vs Cluster 3 | 1.72 | 0.36 | <0.001 | 0.88 – 2.57 |
| **WAIS perceptual reasoning subtest scaled score** | Cluster 1 vs Cluster 2 | 3.15 | 0.43 | <0.001 | 2.14 – 4.16 |
|  | Cluster 1 vs Cluster 3 | 4.78 | 0.39 | <0.001 | 3.85 – 5.71 |
|  | Cluster 2 vs Cluster 3 | 1.63 | 0.36 | <0.001 | 0.77 – 2.48 |
| **WAIS verbal reasoning subtest scaled score** | Cluster 1 vs Cluster 2 | 3.71 | 0.41 | <0.001 | 2.74 – 4.69 |
|  | Cluster 1 vs Cluster 3 | 5.26 | 0.38 | <0.001 | 4.36 – 6.15 |
|  | Cluster 2 vs Cluster 3 | 1.54 | 0.35 | <0.001 | 0.72 – 2.36 |
| **WAIS prorated sum of scaled scores** | Cluster 1 vs Cluster 2 | 37.76 | 3.78 | <0.001 | 28.84 – 46.68 |
|  | Cluster 1 vs Cluster 3 | 55.19 | 3.47 | <0.001 | 47.02 – 63.36 |
|  | Cluster 2 vs Cluster 3 | 17.43 | 3.18 | <0.001 | 9.93 – 24.93 |
| **Theory of Mind z score** | Cluster 1 vs Cluster 2 | -0.34 | 0.14 | 0.036 | -0.66 - -0.02 |
|  | Cluster 1 vs Cluster 3 | 1.02 | 0.13 | <0.001 | 0.72 – 1.31 |
|  | Cluster 2 vs Cluster 3 | 1.36 | 0.11 | <0.001 | 1.09 – 1.63 |
| **Emotion recognition z score** | Cluster 1 vs Cluster 2 | -0.12 | 0.14 | 0.707 | -0.43 – 0.21 |
|  | Cluster 1 vs Cluster 3 | 1.18 | 0.12 | <0.001 | 0.89 – 1.47 |
|  | Cluster 2 vs Cluster 3 | 1.29 | 0.11 | <0.001 | 1.02 – 1.56 |

*Cluster 1: cognitively intact, Cluster 2: moderately impaired, Cluster 3: severely impaired.

Table 8. Post-hoc analyses for significant differences between clinical clusters.

| **Variable** | **Comparison*** | **Mean Difference** | **Standard**  **error** | **p-value** | **95% CI** |
| --- | --- | --- | --- | --- | --- |
| **Positive symptoms** | Cluster 1 vs Cluster 2 | -0.10 | 1.15 | 0.996 | -2.82 – 2.63 |
|  | Cluster 1 vs Cluster 3 | 4.48 | 0.69 | <0.001 | 2.84 – 6.12 |
|  | Cluster 2 vs Cluster 3 | 4.58 | 1.13 | <0.001 | 1.91 – 7.25 |
| **Negative symptoms** | Cluster 1 vs Cluster 2 | -12.91 | 1.06 | <0.001 | -15.42 - -10.41 |
|  | Cluster 1 vs Cluster 3 | 1.37 | 0.64 | 0.083 | -0.14 – 2.88 |
|  | Cluster 2 vs Cluster 3 | 14.28 | 1.04 | <0.001 | 11.82 – 16.74 |
| **BDI-II mood score** | Cluster 1 vs Cluster 2 | 15.76 | 1.71 | <0.001 | 11.73 – 19.79 |
|  | Cluster 1 vs Cluster 3 | 19.57 | 1.03 | <0.001 | 17.14 – 21.99 |
|  | Cluster 2 vs Cluster 3 | 3.81 | 1.67 | 0.061 | -0.14 – 7.76 |
| **Social function** | Cluster 1 vs Cluster 2 | 0.48 | 0.45 | 0.531 | -0.57 – 1.53 |
|  | Cluster 1 vs Cluster 3 | -0.84 | 0.27 | 0.006 | -1.48 - -0.20 |
|  | Cluster 2 vs Cluster 3 | -1.32 | 0.44 | 0.008 | -2.35 - -0.29 |
| **Logical memory scaled score** | Cluster 1 vs Cluster 2 | 2.60 | 0.90 | 0.012 | 0.48 – 4.72 |
|  | Cluster 1 vs Cluster 3 | 0.08 | 0.57 | 0.990 | -1.27 – 1.43 |
|  | Cluster 2 vs Cluster 3 | -2.52 | 0.87 | 0.012 | -4.58 - -0.47 |
| **WAIS perceptual reasoning subtest scaled score** | Cluster 1 vs Cluster 2 | 2.20 | 0.85 | 0.029 | 0.18 – 4.22 |
|  | Cluster 1 vs Cluster 3 | -0.39 | 0.53 | 0.745 | -1.64 – 0.86 |
|  | Cluster 2 vs Cluster 3 | -2.59 | 0.83 | 0.006 | -4.55 - -0.63 |
| **WAIS verbal reasoning subtest scaled score** | Cluster 1 vs Cluster 2 | 2.74 | 0.84 | 0.004 | 0.76 – 4.73 |
|  | Cluster 1 vs Cluster 3 | 0.29 | 0.53 | 0.851 | -0.97 – 1.55 |
|  | Cluster 2 vs Cluster 3 | -2.46 | 0.81 | 0.008 | -4.38 - -0.53 |
| **WAIS prorated sum of scaled scores** | Cluster 1 vs Cluster 2 | 26.59 | 8.36 | 0.005 | 6.82 – 46.37 |
|  | Cluster 1 vs Cluster 3 | -1.17 | 5.19 | 0.972 | -13.46 – 11.12 |
|  | Cluster 2 vs Cluster 3 | -27.76 | 8.12 | 0.002 | -46.98 - -8.54 |
| **Emotion recognition z score** | Cluster 1 vs Cluster 2 | 0.98 | 0.27 | 0.001 | 0.34 – 1.62 |
|  | Cluster 1 vs Cluster 3 | 0.37 | 0.18 | 0.112 | -0.06 – 0.81 |
|  | Cluster 2 vs Cluster 3 | -0.61 | 0.26 | 0.055 | -1.23 – 0.01 |

*Cluster 1: predominant mood symptoms, Cluster 2: predominant negative symptoms, Cluster 3: mild symptoms.

**S3. Results of exploratory cluster analysis to investigate clusters based on both cognitive and clinical variables combined.**

The final cluster solution comprised three distinct groups that followed similar patterns from the cognitive and clinical cluster analyses. The first cluster was characterised by the most severe cognitive impairment and highest level of negative symptoms (N=50), the second cluster was characterised by moderate cognitive impairment and overall moderate symptom severity (N=33), and the final cluster was characterised by intact cognitive function, the lowest level of negative and positive symptoms, and higher mood symptoms (N=53) (See supplementary table 9). At final follow-up, the intact cognitive function/ low psychotic symptom severity group had the highest percentage (53%) of participants who reached clinically significant functional recovery (See supplementary table 10).

General linear mixed model analysis (GLMM) showed no significant effect for cognitive and clinical combined clusters on either social or occupational function.

Table 9. Baseline sociodemographic, clinical, functioning and cognitive characteristics for cognitive and clinical combined clusters.

|  | Cluster 1 moderate cognitive impairment and moderate symptoms (N=53) | Cluster 2 intact cognitive function, high mood symptoms and low negative symptoms (N=33) | Cluster 3 severe cognitive impairment and high negative symptoms (N=50) | ANOVA/ Chi-  Squared |
| --- | --- | --- | --- | --- |
| Demographic |  |  |  |  |
| Male sex (n/ %) | 31 (58.5%) | 22 (66.7%) | 39 (78%) | *χ2*=4.49, *p*=0.106 |
| Mean age (years; mean/SD) | 27.0 (8.5) | 26.3 (5.5) | 26.7 (7.0) | *F*=0.10, *p*=0.903 |
| Clinical |  |  |  |  |
| DUP in days (median, range) | 61 (2920) | 11 (1644) | 35 (5325) | *F*=0.22, *p*=0.801 |
| Positive symptom severity (mean/SD) | 13.1 (4.0) | 12.3 (4.3) | 13.3 (5.2) | *F*=0.50, *p*=0.611 |
| Negative symptom severity (mean/SD) | 14.7 (5.2) | 12.1 (3.4) | 17.6 (7.6) | *F*=8.97, *p*<0.001 |
| BDI-II mood score (mean/SD) | 19.8 (12.0) | 22.8 (10.0) | 15.2 (11.7) | *F*=4.75, *p*=0.010 |
| Functioning (mean/SD) |  |  |  |  |
| Social function | 5.9 (1.6) | 6.5 (1.7) | 5.5 (1.9) | *F*=3.25, *p*=0.042 |
| Occupational function | 4.5 (2.5) | 5.0 (2.9) | 3.1 (2.2) | *F*=5.95, *p*=0.003 |
| Cognition (mean/SD) |  |  |  |  |
| Logical memory scaled score | 7.2 (3.6) | 9.5 (3.3) | 5.5 (2.8) | *F*=15.14, *p*<0.001 |
| WAIS perceptual reasoning subtest scaled score | 9.1 (1.8) | 13.0 (2.0) | 6.1 (1.7) | *F*=146.64, *p*<0.001 |
| WAIS verbal reasoning subtest scaled score | 8.6 (2.0) | 12.1 (2.4) | 5.5 (1.8) | *F*=106.71, *p*<0.001 |
| WAIS prorated sum of scaled scores | 97.5 (10.7) | 138.3 (15.6) | 63.9 (14.5) | *F*=305.43, *p*<0.001 |
| Theory of Mind z score | 0.2 (0.9) | 0.5 (0.7) | -0.1 (0.8) | *F*=6.06, *p*=0.003 |
| Emotion recognition z score | 0.2 (1.1) | 0.7 (0.7) | -0.2 (1.1) | *F*=7.16, *p*=0.001 |

Table 10. Social and occupational functioning across timepoints for clinical and cognitive combined clusters.

|  | **Social function** | | | **Occupational function** | | |
| --- | --- | --- | --- | --- | --- | --- |
|  | **% of cluster in fully functioning category at baseline** | **% of cluster in fully functioning category by 9M** | **% of cluster in fully functioning category by final follow up** | **% of cluster in fully functioning category at baseline** | **% of cluster in fully functioning category by 9M** | **% of cluster in fully functioning category by final follow up** |
| **Cluster 1**  **Moderate cognitive impairment and moderate symptom severity** | 36.5% (19/52) | 50% (22/44) | 47.2% (17/36) | 20.4 % (10/49) | 50% (19/38) | 38.7% (12/31) |
| M(SD) | 5.9 (1.6) | 6.4 (1.6) | 6.1 (1.7) | 4.3 (2.4) | 5.9 (2.8) | 5.3 (2.5) |
| **Cluster 2**  **Intact cognitive function and low psychotic symptom severity** | 51.5% (17/33) | 45.5% (10/22) | 47.6% (10/21) | 25.8 % (8/31) | 43.8 % (7/16) | 53.3% (8/15) |
| M(SD) | 6.1 (1.6) | 6.1 (1.7) | 6.4 (1.5) | 4.0 (2.6) | 6.0 (3.0) | 6.6 (2.7) |
| **Cluster 3**  **Severe cognitive impairment and negative symptom severity** | 32% (16/50) | 31.8 % (14/44) | 26.8% (11/41) | 13% (6/46) | 18.9% (7/37) | 24.2% (8/33) |
| M(SD) | 5.6 (1.9) | 5.8 (1.7) | 5.7 (1.6) | 2.9 (1.8) | 4.5 (2.1) | 4.3 (2.3) |

Table 11. Correlations between premorbid adjustment and clinical, functioning and cognitive variables in SUPEREDEN sample.

| **Variables** | **Baseline PAS total score Early Adolescence** | **Baseline PAS total score Late Adolescence** |
| --- | --- | --- |
| Baseline PAS total score Late Adolescence | 0.46** |  |
| DUP | 0.09 | 0.07 |
| Duration of illness | 0.12 | 0.08 |
| Baseline positive symptom severity | 0.02 | 0.21* |
| Baseline negative symptom severity | -0.04 | -0.07 |
| Baseline social function | 0.04 | 0.02 |
| Baseline occupational function | -0.02 | -0.07 |
| Follow-up social function | 0.20* | 0.19 |
| Follow-up occupational function | 0.11 | 0.22* |
| Baseline logical memory scaled score | 0.03 | 0.05 |
| Baseline WAIS perceptual reasoning subtest scaled score | 0.05 | 0.03 |
| Baseline WAIS verbal reasoning subtest scaled score | -0.04 | -0.04 |
| Baseline Theory of mind score | 0.00 | 0.03 |
| Baseline Emotion recognition score | 0.06 | -0.02 |
| **correlations significant at the 0.01 level  *correlations significant at the 0.05 level  PAS=Premorbid Adjustment Scale | | |

Table 12. Correlations between premorbid cognitive function and clinical, functioning and cognitive variables in SUPEREDEN sample.

| **Variables** | **Test of Premorbid Function total standardised score** |
| --- | --- |
| DUP | 0.23 |
| Duration of illness | 0.27* |
| Baseline positive symptom severity | -0.02 |
| Baseline negative symptom severity | 0.09 |
| Baseline social function | -0.17 |
| Baseline occupational function | -0.04 |
| Follow-up social function | -0.22 |
| Follow-up occupational function | -0.01 |
| Baseline logical memory scaled score | 0.03 |
| Baseline WAIS perceptual reasoning subtest scaled score | 0.42** |
| Baseline WAIS verbal reasoning subtest scaled score | 0.62** |
| Baseline Theory of mind score | 0.47** |
| Baseline Emotion recognition score | 0.39** |
| **correlations significant at the 0.01 level  *correlations significant at the 0.05 level | |
